# Oocyte insemination with the Walking Egg simplified IVF culture system – an investigation into reduced sperm numbers, sperm DNA fragmentation and reactive oxygen species formation

**Published:** 2018-12

**Authors:** GM Boshoff, W Ombelet, C Huyser

**Affiliations:** Department Obstetrics and Gynaecology, University of Pretoria, Private Bag X323, Arcadia, Pretoria, South Africa 0007;; Department Obstetrics and Gynaecology, Genk Institute for Fertility Technology, Genk, Belgium;; UHasselt, Faculty of Medicine and Life Sciences, LCRC, Diepenbeek, Belgium.

**Keywords:** Affordable IVF, insemination, Reactive Oxygen species, DNA fragmentation

## Abstract

**Research question:**

What is the lowest number of sperm that can be used for oocyte insemination during either conventional or the Walking Egg simplified IVF? Does the use of low numbers of sperm in high volume (1 ml) culture media have an effect on sperm DNA fragmentation and reactive oxygen species formation? Also, does the extended co-incubation of embryos with sperm and cumulus cells in the Walking Egg culture tubes induce higher levels of reactive oxygen species?

**Design:**

Binding of sperm to the zona pellucida was compared using a modified hemi-zona assay. In the first part of the study, the binding capacity of decreasing concentrations of motile spermatozoa was evaluated, followed by a comparison of sperm binding after simulated insemination by conventional or the Walking Egg simplified culture protocol. Sperm DNA fragmentation was determined between test and control samples in the second part of the study and reactive oxygen species was measured in spent culture media. As a supplementary examination, reactive oxygen species formation, with the simulated co-incubation of cumulus and sperm cells, was compared between the conventional and Walking Egg IVF culture systems.

**Results:**

Sperm-zona binding in 50 μl culture media, indicated mean sperm binding of more than 20 sperm per hemi-zona with as low as 1000 sperm used for insemination. Using a higher volume of culture media, as is done in the Walking Egg simplified IVF culture system, resulted in 42.8% reduced sperm-zona binding. No significant difference in DNA integrity was observed between the two test groups. The amount of ROS generated during conventional IVF in the first 18 hours of incubation was more than that produced in the simplified culture system over sixty-six hours. Only during extended culture for 114 hours in the simplified culture system, did the ROS generated slightly surpass that of conventional IVF at 18 hours.

**Conclusion:**

Oocyte insemination with as little as 2 x 10^3^ motile sperm showed sufficient sperm-zona binding capacity to be indicative of fertilization potential, supporting the Walking Egg simplified IVF insemination protocol. No difference in DNA fragmentation was observed between conventional and the simplified IVF culture systems, while reactive oxygen species formation was indicated to be at a slower rate during incubation with the Walking Egg simplified IVF culture system than with conventional IVF.

## Introduction

Colloquially, a single sperm is considered to be sufficient to fertilize an egg. In nature and in the assisted reproduction laboratory, however, thousands of sperm are needed to provide hyaluronic acid for enzymatic digestion of bonds between cumulus oophorus cells and the oocyte (Evans, 2002; Megnagi et al., 2015; Primakoff and Myles, 2002; Yin et al., 2009). This enables some sperm to bind to and transverse the zona pellucida, until a single sperm can fertilize the oocyte (Evans, 2002).

Currently there is no universal protocol in practice for conventional in vitro fertilization (IVF) insemination procedures to specify the exact number of sperm needed for this process (Elder et al., 2010; Gardner et al., 2012; Zhang et al., 2013). According to the European Society of Human Reproduction and Embryology’s revised guidelines for good practice in IVF laboratories (2015), “the number of progressively motile sperm used for insemination must be sufficient to optimize the chance of normal fertilization”. This document furthermore states that it is common practice to use approximately 0.1 and 0.5 × 10^6^ progressively motile spermatozoa per millilitre, which equates to insemination with ~20 - 100 x 10^3^ progressively motile sperm in a 200 μl micro-drop of culture media (De los Santos et al., 2016). On the other hand, The Walking Egg (tWE) simplified IVF culture system recommends a protocol of ≤10 x 10^3^ motile sperm per culture tube containing 1 ml culture media, with continued co-culture of fertilized oocytes, sperm and cumulus cells (Klerkx et al., 2014; Van Blerkom et al., 2014).

Comparing the tWE simplified culture system to conventional IVF, major differences such as (i) oocyte insemination with a lower number of progressively motile spermatozoa, (ii) culture with a higher volume of medium, (iii) continuously exposing oocytes/embryos to culture media containing sperm and cumulus cells for a longer incubation time, and (iv) that the culture environment is sealed from the ambient environment can be discerned (Klerkx et al., 2014; Van Blerkom et al., 2014). These differences between IVF culture systems may affect not only oocyte fertilization, but also future embryo development, due to the impact of reactive oxygen species (ROS) formation and deoxyribonucleic acid (DNA) fragmentation of sperm during the fertilization and culture process. On the one hand, ROS are needed for certain steps of development, such as the capacitation and acrosome reaction of sperm (Agarwal et al., 2015), and may even be implicated in the process of oocyte maturation (Guerin et al., 2001; Tiwari et al., 2016). Alternatively, high levels of ROS have been connected with lower oocyte fertilization rates, decreased embryo development resulting in embryos with uneven divisions and increased amount of fragmentation and also decreased clinical pregnancy rates (Agarwal et al., 2015; Guerin et al.; 2001, Yang et al., 1998). Also, lipid peroxidation in sperm by ROS is associated with reduced motility and oocyte-binding capacity, as well as increased sperm DNA fragmentation (Aitken, 2011). Sperm DNA fragmentation, especially when associated with double stranded DNA breaks, has been identified as playing a significant role in decreased embryo development and subsequent implantation (Ribas-Maynou and Bennet, 2019; Santi et al., 2018).

In this study, the tWE simplified IVF culture system’s oocyte insemination and culture protocol are assessed, together with the culture system’s influence on ROS generation and sperm DNA fragmentation. Exploring alternatives for men with moderate to severe male factor infertility, for treatment in clinics where intra-cytoplasmic sperm injection (ICSI) is not available, is paramount.

## Methods

### Human gametes

Gametes were collected from couples seeking assisted reproduction treatment at the Reproductive Biology Laboratory (RBL), Steve Biko Academic Hospital, Pretoria, South Africa, after being formally informed and given an information leaflet requesting their consent for the use of gametes destined to be discarded. The ethical approval for this project was granted by the University of Pretoria Ethics committee (reference number 460/2015). Non-viable unfertilized oocytes (n=123) remaining from ART cycles at the RBL were utilised for hemi-zona binding assay (HZA) testing (Franken et al., 1989). Likewise, human sperm, from semen samples (n=5) processed by density gradient centrifugation and a single wash (PureSperm^®^ 40, 80 & Wash, Nidacon International AB, Mölndal, Sweden), was used for the HZA and DNA fragmentation testing. Culture media (Global^®^ Total^®^ for Fertilization, LifeGlobal, Guilford, Connecticut) used during experimentation was employed in the measurement of ROS.

### Experimental design

**Figure 1:**
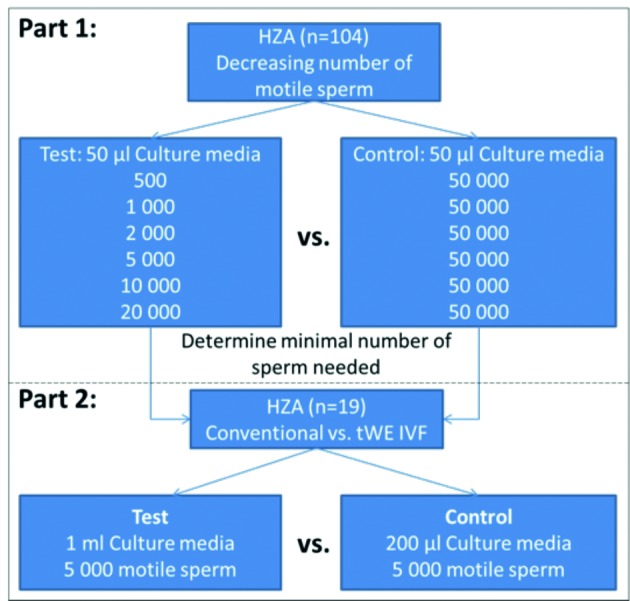
Flow diagram depicting parts 1 and 2 of hemi-zona binding assay. Part 1: Decreasing consentrations of motile sperm within the same volume of culture media are compared. Part 2: The same number of motile sperm in two different volumes of culture media are evaluated.

This study was initiated with the evaluation of decreasing concentrations of spermatozoa by a HZA. Following this, a direct comparison of sperm binding, via HZA, between conventional IVF and tWE simplified culture protocol after simulated insemination was performed. Non-viable, unfertilized bisected oocytes (n=104 and n=19, respectively) were used. During the first part of the study, each hemi-zona was placed in a 50 μl micro-drop of pre-gassed culture media, covered by mineral oil (FertiCult^TM^ - Mineral Oil, FertiPro NV, Beernem, Belgium), along with either 50 x 10^3^ (control) or a decreased number (test), ranging from 0.5 x 10^3^ to 20 x 10^3^, motile spermatozoa (Figure 1). In the second part of the study, each hemi-zona placed in either a 200 μl culture drop (control) or in a tWE culture tube (test) containing 1 ml gassed culture media, as performed in the simplified culture system and inseminated with 5 x 10^3^ motile spermatozoa (resulting in 5 x 10^3^ and 25 x 10^3^ motile spermatozoa per millilitre, respectively) (Figure 1). The samples were incubated for 18 hours in a conventional embryo culture incubator (7.35% CO2, 5% O2; K-Minc^TM^, Cook Medical, Bloomington, Indiana) (control) and laboratory warm bath (DB-006, K-Systems Kivex Biotec A/S, Birkerod, Denmark) (tWE culture tube), set at 37°C. For each group, the hemi-zonae were removed from the culture media and the number of spermatozoa bound was counted to determine the minimal number of spermatozoa required to inseminate when employing the simplified tWE culture system.

### Sperm Hemi-zona binding assay

The hemi-zona binding assay (HZA) bio-test was performed according to Franken et al., (1989). Briefly, oocytes were bisected using a micromanipulator (Transferman NK2, Eppendorf, Hamburg, Germany) with a micro-blade (BD Micro-Sharp^TM^, Beckton, Dickinson and Company, Franklin Lakes, NJ). From a single oocyte, one of the two halves of the zona pellucida was allocated to the test and its counterpart to the control group. The two hemi-zonae were placed in the relevant (test vs. control) culture media and washed spermatozoa were introduced.

Insemination of hemi-zonae in micro-drops was performed by diluting processed sperm with Puresperm^®^ Wash, to appropriate concentrations so that a 5 to 10 μl volume would contain the prerequisite number of motile spermatozoa. The exact volume needed was then calculated and pipetted into the micro-drops containing hemi-zonae using a calibrated variable volume pipette (Finnpipette^®^ F2).

For insemination into the tWE glass tubes, a single drop of the diluted sperm sample was expelled into a pre-equilibrated tube, using a 1 ml syringe and an 18 gauge needle. To ensure that accurate insemination counts were injected in the tubes, increasing volumes of water (5 – 100 μl), with trypan blue (Cat. no. 15250061, 0.4% Trypan Blue Solution, Thermo Fisher Scientific Inc., Waltham, Massachusetts) added for improved visualization, were pipetted onto a petri dish (Cat. no. 150360, 90 mm diameter NuncTM IVF Petri Dish, Thermo Fisher Scientific Inc., Waltham, Massachusetts), using a calibrated variable volume pipette. Alongside these drops, additional drops were made by expelling either a single or two drops of the coloured water from a 1 ml syringe with an 18 or 26 gauge needle (Cat. no. 305196 18 G & 30511 26 G, BD PrecisionGlide^TM^) attached. By visual comparison, a single drop of fluid expelled from an 18 gauge needle was determined to be equivalent to 25 μl in volume. Having established the volume being inseminated, sperm samples were diluted to an appropriate concentration, as to have 5 x 10^3^ motile sperm in 25 μl. The hemi-zonae and sperm were incubated for 18 hours in either a humidified conventional embryo culture incubator (micro-drops) or a warming bock at 37°C (tWE tubes), to simulate the time oocytes and sperm are co-incubated before the first pre-zygote evaluation.

To observe and accurately count the number of spermatozoa bound to the hemi-zonae, the hemi-zonae were incubated for one hour at room temperature in phosphate buffered saline (PBS; P4417, Sigma-Aldrich Pty Ltd, Johannesburg, South Africa) containing 0.02 M glycine. Following incubation, the hemi-zonae were washed in PBS and placed in 4 μM ethidium homodimer (EthD-2; LIFE Technologies, Johannesburg, South Africa) for 30 minutes (in the dark at room temperature). After staining, the hemi-zonae were washed (x3) in PBS and mounted on glass coverslips (22 x 22 mm microscope cover glass, Lasec SA, Johannesburg, South Africa), held by 1 μl droplets of clear nail polish on each corner over a microscope slide (76 x 26 x 1 mm, Lasec SA, Johannesburg, South Africa), using antifade mounting medium (Prolong Diamond Antifade 5; LIFE Technologies, Johannesburg, South Africa).

Subsequently, the minimum sperm concentration, which would provide sufficient sperm binding, was determined from counts of spermatozoa bound to hemi-zonae using a confocal laser-scanning microscope (LSM 510 Meta confocal, Zeiss, Oberkochen, Germany). For each hemi-zona, double-blinded counts, by two experienced evaluators were perfomed at 400 times magnification (Axiovert 200; Zeiss, Oberkochen, Germany). If fewer than 50 spermatozoa were bound to a control hemi-zona the corresponding sample and data-set was discarded.

### Sperm deoxyribonucleic acid packaging

Individual DNA packaging of spermatozoa remaining in the culture media was assessed using a toluidine blue stain (Marchesi et al., 2010; Sasikumar and Dakshayani, 2013). Due to low volumes and numbers of sperm (200 μl culture media and 5 x 10^3^ motile sperm per sample), samples (n=36) were pooled by combining three samples from the same group (test or control, n=12 pooled samples) before sperm and culture media were separated by centrifugation (Centrifuge 5417R, Eppendorf, Hamburg, Germany) at 500g for 10min. After centrifugation, 10 μl sperm pellets were removed by careful pipetting, smeared onto a glass microscope slide and left to air dry. Sperm samples were then fixed to the slides by 30 minute exposure to a 1:1 mixture of 96% ethanol and acetone and allowed to air dry. Fixed slides were counterstained stained with 0.1% nigrosine and placed in 0.1M HCl for 15 minutes to hydrolyse the spermatozoa and then rinsed twice with distilled water. Slides were finally submerged in a 0.05% toluidine blue solution for 15 minutes, removed and left to air dry (Gardner et al., 2012).

Evaluation of stained spermatozoa was performed using a light microscope (Axioskop 40, Zeiss, Germany) and 40x-phase contrast objective. A total of 200 spermatozoa per sample were counted, to distinguish lightly stained (normal DNA packaging) from darker stained sperm (abnormal DNA packaging or DNA fragmentation present), and the percentage DNA fragmentation calculated (Marchesi et al., 2010).

### Reactive oxygen species generation in the culture media

Following the DNA fragmentation evaluation, supernatants from all samples, after the removal of spermatozoa, were frozen for subsequent ROS evaluation. The concentration of ROS in each (control and test) sample was evaluated using the fluorogenic probe 2’,7’-dichlorodihydrofluorescein diacetate (DCF-DA; C24H16Cl2O7; D6883,Sigma-Aldrich Pty Ltd, Johannesburg, South Africa). In addition, to evaluate the contribution of cumulus cells on ROS generation during incubation, tWE culture tubes (n=6) with 1 ml culture media and conventional IVF insemination dishes with 250 μl culture media drops (n=6) were prepared and gassed. Excess, discarded cumulus cells, removed during a standard oocyte aspiration were cut into approximately 10 x 10 x 5 mm sections. Individual pieces were then placed in pre-equilibrated culture media with either 5 x 10^3^ motile spermatozoa (tWE culture tubes) or 50 x 10^3^ motile spermatozoa (conventional IVF insemination droplets). The culture tubes were kept in a warming block at 37°C for the duration of the experiment and the insemination dishes kept in a conventional embryo culture incubator (7.35% CO_2_, 5% O_2_, 37°C).

Prior to the initiation of culture, 50 μl culture media was removed from all culture tubes and insemination drops. Incubation then ensued, with another 50 μl of media removed after 18 hours of culture. During conventional IVF, fertilized oocytes would be removed from the insemination media after approximately 18 hours of culture (De los Santos et al., 2016; Gardner et al., 2012). Therefore, incubation of the insemination dishes were terminated after 18 hours and no more sampling from these dishes was performed. The culture tubes simulating the simplified tWE IVF culture system were cultured up to 114 hours after insemination, with 50 μl culture media samples being removed from each culture tube at 66 and 114 hours after insemination. Culture media samples were removed from the culture tubes using a needle and syringe, without opening the tubes. According to manufacturer’s specifications (OxiSelect^TM^ In Vitro ROS/RNS Assay Kit, Cell Biolabs, Inc., San Diego, CA), all samples were centrifuged at 1 x 10^4^ G for 5 minutes to eliminate any insoluble particles and the supernatant removed for storage in 2 ml Cryovials^TM^ at -196°C in order to batch samples for evaluation.

A fresh H_2_O_2_ standard control (H1009, Sigma-Aldrich Pty Ltd, Johannesburg, South Africa) was prepared on the day of experimentation by diluting a stock concentration of 30 μM H_2_O_2_ in Global^®^ Total^®^ for Fertilization in a stepwise fashion by the removal of 500 μl of the stock solution, to which 1000 μl Global^®^ Total^®^ for Fertilization was added. Hereafter, the process was repeated until concentrations of 30, 10, 3.333, 1.111, 0.370, 0.123, 0.041 and 0.014 μM H_2_O_2_ were obtained. Test samples were thawed to room temperature just prior to the readings being performed.

A working solution of 10 μM DCF-DA in PBS was freshly prepared before experimenting and 50 μl DCF-DA, together with 50 μl volume of either sample (n=6 repeats per test sample) or H_2_O_2_ concentration (n=3 repeats per concentration) was pipetted into separate wells of a sterile, untreated, white bottomed, 96-well plate (Nunc 236105, Thermo Scientific, Roskilde, Denmark) to have a final volume of 100 μl in each well. Negative controls of Global^®^ Total^®^ for Fertilization, sterile water and 10 μM DCF-DA (n=2 repeats each) were also prepared. The 96-well plate was placed in a multimode plate reader (Biotek Synergy 2, Biotek Instruments, Winooski, Vermont). Excitation was performed at 485 nm, and fluorescence emission at 590 nm was detected, according to manufacturer’s specifications (OxiSelect^TM^ In Vitro ROS/RNS Assay Kit).

### Statistical analysis

A one-way analysis of variance with Bonferroni adjustment was employed to compare sperm-zonae binding numbers in six tests and one control group after HZA with decreasing sperm numbers. Descriptive statistics were used to determine 99% confidence intervals for insemination groups of interest in respect of sperm-zonae binding. The derived minimum insemination number was used to compare two culture methods by HZA. A paired t-test was performed to compare the association between sperm-zona binding in the test and control groups with a 0.05 level of significance. Assessments of sperm and culture media remaining after the completion of the previous experiment were used to evaluate sperm DNA fragmentation and culture media ROS generation. Wilcoxon’s signed-rank test compared test vs. control groups in both cases at a 0.05 level of significance.

## Results

### Minimal sperm insemination numbers

**Figure 2:**
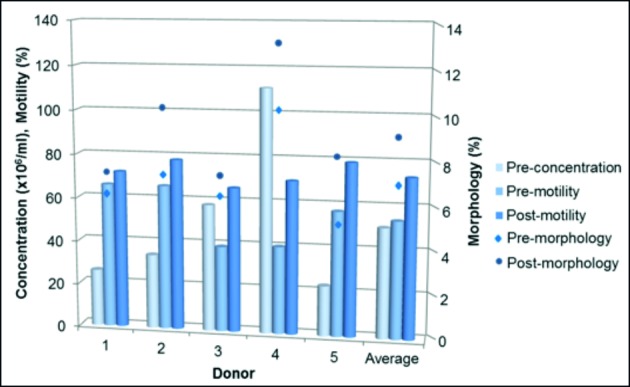
Individual and average sperm parameters (bar chart on primary axis: concentration and motility; X-Y scatter plot on secondary axis: morphology) of donors (n=5) used during the hemi-zona assay.

Binding of sperm to the zona pellucida was compared by a modified HZA, using semen samples from five donors that had sperm parameter values above the lower reference limits [5th edition of the World Health Organization’s Laboratory manual for the examination and processing of human semen (2010)]. Sperm parameters can be seen in Figure 2. Sperm-zona binding (mean ± SD & 95% CI), per group of sperm insemination number, is listed in Table I.

**Table I:** Spermatozoa mean binding to hemi-zonae after insemination of sperm (95% CI, n=104).

Sperm insemination number (x10^3^/ml)	Mean no sperm bound	(95% CI)
0.5	13.948	(9.78 ; 18.12)
1	20.533	(16.21 ; 24.85)
2	35.314	(28.14 ; 42.49)
5	39.638	(32.06 ; 47.22)
10	62.824	(50.68 ; 74.97)
20	73.256	(61.92 ; 84.59)
50	119.720	(105.08 ; 134.36)

### Conventional culture versus the simplified tWE culture

**Figure 3:**
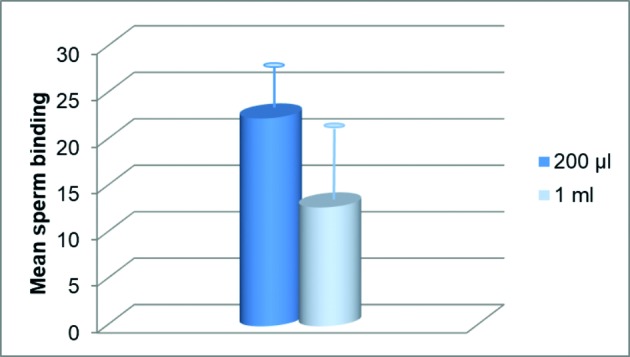
Mean sperm binding (±SD) to hemi-zonae in 200 μl vs. 1 ml culture media after insemination with 5 x 10^3^ motile sperm (n=19); p=0.003.

Sperm hemi-zonae binding in 200 μl and 1 ml culture media, after insemination with 5 x 10^3^ motile sperm, resulted in a mean (±SD) sperm binding per hemi-zona of 22.36 ± 5.06 and 12.79 ± 7.75 for the two groups, respectively (Figure 3). The difference between the two groups (p=0.003) was calculated as 42.8% with 95% CI of [31.71%; 82.58%].

### Sperm deoxyribonucleic acid packaging

**Figure 4:**
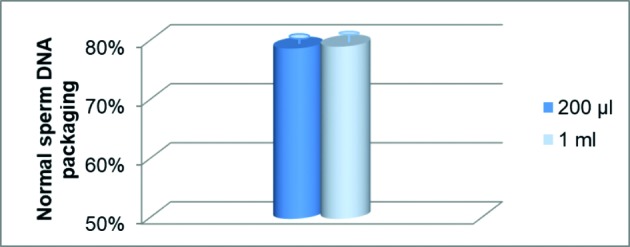
Mean (±SD) sperm DNA integrity after insemination in 200 μl and 1 ml culture media (n=12); p=0.901.

The mean (±SD) percentage of sperm with normal DNA packaging from the two culture media volumes evaluated were 78.8% ± 2.71 and 79.2% ± 4.02 (Figure 4). No significant difference in DNA integrity was observed between the two test groups.

### Reactive oxygen species generation

The fluorescence measured in 200 μl and 1 ml culture media, after insemination with 5 x 10^3^ sperm, was (mean RFU ± SD) 40735 ± 195 and 40796 ± 693, respectively, with no significant difference observed. The simulated conventional and tWE simplified IVF cultures system’s mean RFU ± SD values, as measured at 0 and 18 hours (conventional and tWE), as well as 66 and 114 hours (tWE only) can be seen in Table II.

**Table II:** Mean fluorescence (RFU ± SD) measured in culture media samples, comparing the simplified Walking Egg (tWE) and conventional in vitro fertilization (IVF) culture systems.

Time (hours)	Fluorescence (RFU ± SD)	
	Conventional IVF	Simplified tWE IVF
0	39938 ±525	39960 ±815
18	40825 ±192	40315 ±716
66	N/A	40659 ±693
114	N/A	41182 ±857

## Discussion

The simplified system could be considered as an innovative alternative to conventional IVF. This system allows for the use of minimalistic numbers of sperm for fertilization and the continuous culture of embryos which are maintained in a single step culture media, together with the remaining sperm and cumulus cells (Van Blerkom et al., 2014). The number of sperm required for insemination is debatable, often leaving men suffering from oligozoospermia with no other option than ICSI (Craft, 1982; De los Santos et al., 2016; Elder et al., 2010; Gardner et al., 2012; Zhang et al., 2013). However, Craft, in 1982, stated that as much as 10 x 10^3^ sperm can affect fertilization and speculated that even less may be sufficient. He also declaring that the removal of fertilized oocytes from the dish containing cumulus cells was unnecessary (Craft, 1982). In this study, a direct comparison between conventional IVF and the tWE simplified system, in regards to their capacity for minimal sperm insemination and its influence in terms of DNA fragmentation and ROS generation was performed.

The binding of 20 sperm to a hemi-zona is considered as a cut-off value indicating that the zona is of acceptable quality for the bio-test, and that the fertilization potential of a sperm sample is adequate (Franken et al., 1991). The results from the current HZA indicated >20 sperm binding from sperm insemination numbers of as few as 1 x 10^3^ motile sperm could be achieved. However, considering the statistical analysis (95% CI lower limit; 16.21 sperm bound for 1 x 10^3^ motile sperm inseminated), groups with >2 x 10^3^ motile sperm used for insemination displayed lower limits of >20 sperm bound per oocyte. Therefore, a minimum cut-off value of 2 x 10^3^ motile sperm for insemination during conventional IVF is recommended.

Also, during experimentation, difficulty was initially experienced with the retrieval of hemi-zonae from the culture tubes (tWE culture system group). Locating the hemi-zonae within micro-droplets is already challenging, as the zonae are transparent glycol-protein structures (Primakoff and Myles, 2002). This proved to be near impossible when attempting to retrieve the hemi-zonae in the large volume of the tWE tubes, achieving the retrieval of only three out of the nineteen hemi-zonae. The experimental design was adapted to improve visualization of the hemi-zonae by replacing the tWE tubes with 4-well dishes. From this, 1 ml culture media containing hemi-zonae were inseminated with 5 x 10^3^ motile spermatozoa and left to incubate for 18 hours in a conventional embryo culture incubator. Hereafter, the hemi-zonae were easily located and all hemi-zonae were retrieved after the test period.

After the volume of culture media had been increased in-line with the simplified tWE IVF culture system protocol, the corresponding reduction in sperm concentration, as expected, resulted in a decline in the number of sperm bound to hemi-zonae. To compensate for this lower binding, the number of sperm to be inseminated could be increased. A minimum cut-off of 2 – 5 x 10^3^ motile sperm for insemination in the simplified tWE IVF culture system is therefore proposed, depending on a holistic consideration of all sperm parameters.

The assessment of sperm DNA packaging by toluidine blue facilitated the indirect measurement of sperm DNA integrity (Sasikumar and Dakshayani, 2013), comparing the two culture systems, yet demonstrating no significant differences in the current study. Since the volume of culture media and the number of sperm used for insemination does not influence sperm DNA fragmentation, the reduced sperm for insemination protocol can be applied with the simplified tWE IVF culture system.

Furthermore, the experiment’s setup simulated the cumulus-oocyte-complex insemination and subsequent continuous culture of embryos in the simplified tWE IVF culture system and during conventional IVF respectively. The extended culture of embryos in media containing cumulus and sperm cells, as is performed in the simplified tWE IVF culture system, could present a potential vulnerability due to a build-up of ROS over time. The cells contained in the culture tube would continue cellular metabolism until programmed and non-programmed cell death occurs, with the metabolic by- and cellular breakdown-products continuously generating ROS (Chaudhari et al., 2014). Therefore, with the extended culture as performed with the simplified tWE IVF culture system, a linear increase in ROS over time can be expected.

Interestingly, upon comparison of the level of ROS generation between the two culture systems evaluated, a lower presence of ROS was displayed in the simplified culture system. The values obtained indicated no significant difference between any of the groups tested, although an observational evaluation of the geometric averages obtained does indicate a trend suggesting that the amount of ROS generated during conventional IVF in the first 18 hours of incubation was more than that produced in the simplified culture system over sixty-six hours (day 3 of culture). Only during extended culture for 114 hours (day 5 of culture) in the simplified culture system, did the ROS generated slightly surpass that of conventional IVF at 18 hours. It is hypothesised that the low numbers of sperm and high culture media volume of the simplified tWE IVF culture system counteract the increased culture time, thereby minimizing ROS exposure of embryos remaining in the culture media in which fertilization occurred.

In closure, oocyte insemination with as little as 2 x 10^3^ motile sperm showed to be sufficient by the HZA, indicative of fertilization potential. This finding endorses the efficiency of the Walking Egg simplified IVF insemination protocol using minimalistic sperm numbers.
